# Pressure-Waveform Guidance for Pulmonary Vein Occlusion During Cryoballoon Ablation: Procedural Efficiency and Repeat-Procedure Findings from a Large Single-Center Cohort

**DOI:** 10.3390/jcm15197462

**Published:** 2026-09-25

**Authors:** Obaida Alothman, Ahmad Keelani, Georgi Borisov, Markus Frommhold, Hytham Abdelwahab, Gabriele D’Ambrosio, Karam Issa, Sultan Alshehri, Santi Raffa, J. Christoph Geller

**Affiliations:** 1 Arrhythmia Section, Division of Cardiology, Zentralklinik, 99437 Bad Berka, Germany; a_kelani@hotmail.com (A.K.); georgi.borisov@zentralklinik.de (G.B.); hytham.abdelwahab@zentralklinik.de (H.A.); karam_issa@hotmail.com (K.I.); hz.alshehri23@gmail.com (S.A.); santi.raffa@zentralklinik.de (S.R.); christoph.geller@zentralklinik.de (J.C.G.); 2 School of Medicine, Otto-von-Guericke University, 39120 Magdeburg, Germany

**Keywords:** pulmonary vein isolation (PVI), cryoballoon (CB), atrial fibrillation (AF), contrast media (CM), fluoroscopy exposure

## Abstract

**Background/Objectives**: The cryoballoon (CB) has simplified pulmonary vein isolation (PVI) for treating atrial fibrillation (AF). Vein occlusion is usually assessed utilizing contrast media which entails possible risks and increases fluoroscopy exposure. Pressure-guided assessment of occlusion has emerged as a potential alternative. The objective of this study is to compare procedural characteristics and repeat-procedure findings of pressure-guided and contrast-guided assessment of vein occlusion. **Methods**: From July 2018 to December 2023, 1166 patients (485 [41%] female, mean age 65 ± 10 years) with AF (64% paroxysmal) undergoing CB-PVI were enrolled at a single-center, 368 (32%) underwent a contrast-guided and 798 (68%) a pressure-guided approach. **Results**: Procedure duration (90 ± 20 vs. 93 ± 23 min) and ablation time (1342 ± 361 vs. 1368 ± 406 s) were similar in pressure- and contrast- guided groups, and the acute success rate of PVI was high in both groups. The pressure-guided approach had shorter X-ray times (5.7 ± 3.4 vs. 9.5 ± 4.8 min, *p* < 0.0001) and lower X-ray doses (82 ± 78.9 vs. 586 ± 546 cGy/cm^2^, *p* < 0.0001). In 112 redo-procedures for arrhythmia recurrence after CB-PVI, durable isolation of all veins was observed more often after pressure-guided procedures (28/56 [50%] vs. 13/56 [23%]); per-vein durability was also higher (174/224 [78%] vs. 150/224 [67%], *p* = 0.015). **Conclusions**: Pressure-waveform guidance during CB-PVI is a feasible alternative to contrast-guided occlusion assessment and was associated with substantially lower intraprocedural fluoroscopy exposure. Repeat-procedure findings suggest a possible durability signal, but this observation should be considered exploratory and confirmed in prospective studies.

## 1. Introduction

Pulmonary vein isolation (PVI) using a cryoballoon (CB) provides efficacy and safety comparable to radiofrequency ablation and has also been shown to be superior to antiarrhythmic drug therapy as an initial rhythm-control strategy in selected patients [1,2,3].

A key determinant of effective CB ablation is adequate occlusion of the pulmonary vein (PV) ostium [4,5,6]. Stable balloon–tissue contact and complete venous occlusion limit convective warming by residual blood flow, thereby facilitating efficient tissue cooling and formation of a continuous transmural lesion. Conversely, incomplete occlusion or early balloon dislodgement may result in non-uniform cooling, ineffective lesion formation, and subsequent PV reconnection.

Pulmonary vein occlusion is traditionally evaluated using contrast media (CM) injection, which has potential disadvantages (e.g., deterioration of renal function, allergic reaction). Moreover, evaluating pulmonary venography can be challenging, often resulting in increased fluoroscopy exposure and added risk for both patients and operators.

Although echocardiographic assessment of vein occlusion (using transesophageal [TEE] or intracardiac echo [ICE]) is feasible [4,7], it demands specialized expertise and increases the complexity of the procedure (e.g., TEE often requires general anesthesia, and prolonged esophageal probe placement may increase the risk of complications. ICE, while effective, involves higher procedural costs and necessitates additional venous access, elevating the risk of vascular complications).

Pressure-waveform analysis offers a simpler physiological method of assessing PV occlusion. Before occlusion, the pressure recorded through the distal balloon lumen reflects left atrial pressure. Complete sealing of the PV ostium produces a characteristic change to a pulmonary artery-like waveform, indicating isolation of the distal PV pressure compartment [8,9,10,11,12]. Pressure monitoring may also identify loss of occlusion during the initial phase of freezing, including early balloon displacement associated with balloon expansion. This approach does not require additional equipment or personnel. In preliminary studies, it has shown accuracy and acute success rates similar to those of TEE-guided procedures [8,9,10,11].

With the exception of a single small study [10], subsequent investigations involving larger patient cohorts [8,13,14] have consistently shown that pressure-guided assessment of pulmonary vein occlusion yields procedural and clinical outcomes comparable to those achieved with the conventional contrast-guided approach.

Several studies have evaluated pressure-waveform analysis for assessing pulmonary vein (PV) occlusion as an adjunct to contrast-medium injection rather than as a standalone method. An exclusively pressure-guided strategy performed without contrast injection has not previously been evaluated in a large cohort. Moreover, no study has directly compared PV durability during repeat electrophysiology study following exclusively pressure-guided versus contrast-guided CB-PVI. To address these gaps, the present study compared both strategies in a large single-center cohort, examining procedural characteristics, fluoroscopy exposure, acute outcomes, and invasively assessed PV durability in a subgroup of patients undergoing repeat ablation for recurrent atrial arrhythmia.

## 2. Materials and Methods

In this single-center study, 1166 consecutive patients with symptomatic paroxysmal or persistent AF undergoing first AF ablation at our institution between July 2018 and December 2023 were collected prospectively in a dedicated database and retrospectively analyzed. Patients who had previously undergone PVI or any other left atrial ablation were not enrolled, as repeat procedures were performed using detailed electroanatomical mapping and a point-by-point ablation strategy. Patients requiring additional ablation (e.g., cavo-tricuspid isthmus [CTI] or AV-node reentry), and procedures aborted due to severe complications (one because of respiratory failure, necessitating rescheduling under general anesthesia, and one because of technical problems) were excluded (Figure 1).

The occlusion-assessment strategy was not randomized and not based on clinical characteristics. Contrast-guided assessment represented the initial institutional practice, whereas pressure-waveform guidance was subsequently introduced (Table 1b) initially by operator preference and later became the routine strategy by all operators. To address potential confounding related to the non-randomized design and unequal group sizes, propensity-score adjustment was performed. Both 1:1 propensity-score matching and inverse-probability weighting substantially improved covariate balance between groups, supporting comparability of the adjusted cohorts (Table 1a).

This study compared the procedural characteristics and repeat-procedure findings of pressure-guided versus contrast-guided PV occlusion assessment during CB-PVI. The primary endpoints were radiation exposure, procedure duration, and CB application time. Radiation exposure was tracked using fluoroscopy time and the dose area product (DAP), both of which were recorded automatically by the system. To determine the effective dose, we multiplied the DAP by established sex-specific conversion factors—0.0020 mSv/(cGy·cm^2^) for men and 0.00276 mSv/(cGy·cm^2^) for women.

Secondary endpoints included acute isolation of all PVs, the need for RF touch-up, and acute procedural complications.

### 2.1. Preparation Before Procedure

Contrast-enhanced computed tomography (CT) was performed to delineate left atrial (LA) anatomy, exclude left atrial appendage thrombus and guide the procedure. CT images were reconstructed into a 3D model integrated into the fluoroscopy system utilizing the EP Navigator (Koninklijke Philips N.V., Amsterdam, The Netherlands) to outline LA and pulmonary vein anatomy, facilitate transseptal puncture by localizing the fossa ovalis, and improve interpretation of anatomic structures on fluoroscopic images. TEE was performed if left atrial appendage (LAA) thrombus could not be adequately excluded by CT.

Patients on vitamin K antagonists (VKA) were maintained at a target international normalized ratio (INR) of 2.0–3.0. For those on direct oral anticoagulants (DOACs), a minimally interrupted oral anticoagulation (OAC) regimen (omitting the morning dose of the drug on the day of the procedure) was employed.

### 2.2. Procedure

Procedures were performed under standardized moderate-to-deep sedation with spontaneous ventilation. Intravenous propofol (mean total dose 947.5 ± 331.4 mg) and fentanyl (57.1 ± 27.1 µg) were administered by trained personnel and titrated to maintain a target MOAA/S (Modified Observer’s Assessment of Alertness/Sedation) score of 1–2. Patients were continuously monitored using ECG, non-invasive blood pressure, oxygen saturation, respiratory rate, and capnography.

Luminal esophageal temperature (LET) was monitored using a temperature probe (S-Cath, Circa Scientific, LLC, Englewood, CO, USA). The esophageal course was reconstructed from the preprocedural CT slices to detect proximity to targeted ablation sites.

Femoral venous access was obtained mainly using ultrasonographic guidance [15,16]. A decapolar diagnostic catheter (IBI-Inquiry^TM^, Abbott, IL, USA) was positioned at the His-bundle, which served as a reference during the transseptal puncture and provided pacing capability when required.

PVI was performed following a single transseptal puncture using a 15F steerable sheath (FlexCath™, Medtronic, Minneapolis, MN, USA), the 28 mm CB (Arctic Front Advance™, Medtronic, Minneapolis, MN, USA), and an inner-lumen circular 8-pole mapping catheter (Achieve™, Medtronic, Minneapolis, MN, USA).

During energy application, pulmonary vein signals were monitored to measure the time-to-isolation (TTI). Routinely, either a single (240 s, if TTI was <60 s) or two (180–240 s) applications were given per vein [17].

The endpoint was bidirectional (entrance and exit) conduction block of all veins.

During ablation of septal veins, continuous phrenic nerve pacing was performed using the decapolar diagnostic catheter (IBI-Inquiry^TM^, Abbott, IL, USA) positioned in the superior vena cava to assess diaphragmatic contractions. In addition, compound motor action potential (CMAP) recordings of diaphragmatic activity were employed to monitor phrenic nerve integrity [18,19,20]. Ablation was immediately stopped if there was any tactile reduction or cessation of diaphragmatic contraction or if CMAP amplitude decreased by >25%.

Heparin was administered intravenously, with activated clotting time (ACT) checks every 20 min to maintain an ACT between 300 and 400 s.

**Pulmonary vein occlusion evaluation:**

In the contrast-guided approach, CM was injected distally via the balloon tip. Fluoroscopy was used to assess vein occlusion and detect any CM leakage; the balloon position was adjusted until optimal occlusion without CM leakage was achieved (Figure 2).

In the pressure-guided approach, LA pressure was continuously measured via the balloon’s distal tip. During sinus rhythm, both atrial (A) and ventricular (V) waves can be observed (Figure 2A). In AF, only the V wave is present due to the absence of consistent atrial contraction (Figure 2C). Under fluoroscopic guidance and using the CT integration, the balloon was advanced to the pulmonary vein ostium in order to obtain a coaxial position of the sheath-CB-circular mapping catheter assembly with the main PV axis. When vein occlusion was achieved, there was an abrupt shift in the pressure waveform to a pulmonary artery-like curve (Figure 2B). This change is evident both in sinus rhythm (Figure 2B) and AF (Figure 2D), confirming complete occlusion, and the freezing process was initiated. Because physiological factors (including respiratory pattern, sedation depth, intravascular volume status, and vasoactive medication use) may influence waveform characteristics, interpretation was based on the morphological change rather than on an absolute pressure threshold.

Early dislodgement (‘pop-out’ phenomenon) due to the slight increase in size and change in conformation of the balloon can be identified by monitoring pressure changes during the first few seconds of freezing, allowing for adjustments of the balloon position. This early catheter displacement can result in suboptimal vein occlusion which may not always be detected when relying solely on contrast medium for vein occlusion assessment [12].

Once the ice ball forms and the inner lumen freezes, pressure visualization ceases. After the freezing process is complete and the ice ball thaws, pressure visualization resumes.

### 2.3. Post-Ablation Care

In all patients, transthoracic echocardiography was conducted following the procedure to exclude pericardial effusion. Femoral hemostasis was achieved using a figure-of-eight suture technique in combination with a pressure bandage, removed 6 h after the procedure. The suture was removed the following morning. All patients were prescribed a combination of proton-pump inhibitors and antacids for 6 weeks. Oral anticoagulation therapy was reinitiated 6 h after the procedure, contingent on the absence of bleeding complications, and maintained for a minimum of 3 months after ablation.

### 2.4. Follow-Up and Repeat Procedures 

Clinical follow-up after the index CB-PVI was structured according to the institutional follow-up protocol, with structured ECG Holter monitoring at 3, 6, and 12 months and annually thereafter. Additional rhythm monitoring was performed whenever patients reported symptoms suggestive of recurrent arrhythmia. Information from external cardiologists or hospitals was included when an interpretable ECG tracing or rhythm-monitoring report was available. Recurrence was defined as documented atrial arrhythmia (including AF, atrial flutter, or atrial tachycardia) lasting >30 s after a 90-day blanking period. Repeat ablation was considered for clinically relevant, documented recurrence based on symptoms, patient preference, and electrophysiologist assessment. However, long-term outcome data were not available in all cases. 

Repeat procedures were performed using a three-dimensional electroanatomic mapping system (EnSite™ Precision or X, Abbott, IL, USA) and a high-density mapping catheter (HD-Grid Advisor™, Abbott, IL, USA). Low voltage areas were defined as regions with bipolar electrogram amplitudes < 0.5 mV [21].

### 2.5. Statistical Analysis

Continuous variables are expressed as mean ± standard deviation [SD], and categorical variables as counts and percentages. Between-group comparisons of continuous variables were performed using the independent-samples Student’s *t*-test, and categorical variables were compared using Pearson’s chi-square test, as appropriate. To address potential confounding related to the non-randomized design and unequal group sizes, propensity-score adjustment was performed using clinically relevant baseline variables. Sensitivity analyses included 1:1 propensity-score matching, inverse-probability-of-treatment weighting, and adjustment for the logit of the propensity score. Covariate balance was assessed using standardized mean differences, with values < 0.10 considered acceptable. Two-sided *p* values < 0.05 were considered statistically significant. Analyses were performed using SPSS Statistics version 24.0 and Python version 3.10.

## 3. Results

### 3.1. Patient Characteristics

The study cohort consisted of 1166 patients (485 [41%] female, mean age 65.4 ± 10.4 years), with 64% having paroxysmal AF. Of these, 368 patients (154 female [41%], age 64.5 ± 10 years) were treated using the contrast-guided approach, and 798 patients (331 female [41%], age 65.7 ± 10 years) using the pressure-guided approach.

Patient characteristics are presented in Table 1a. The chronological distribution of procedures according to the pulmonary vein occlusion-assessment strategy is shown in Table 1b.

Patients in the pressure-guided group had a higher rate of persistent AF and a higher CHA2DS2-VASc score; no additional differences were observed between groups.

PV anatomical variants included a left common PV in 101 patients (8.6%), an additional right middle PV in 21 patients (1.8%), and an inferior common PV in one patient. One additional patient had an absent right inferior PV following lobectomy of the lung.

### 3.2. Procedural Data

Table 2a presents procedural outcomes and comparisons adjusted for baseline patient characteristics. Both IPTW and propensity-score matching showed that pressure-guided procedures were associated with shorter fluoroscopy time and lower radiation exposure. The small reduction in procedure duration observed with IPTW was borderline and was not confirmed in the matched analysis. Neither approach demonstrated a clear difference in ablation time.

Median intraprocedural contrast volume was 50 mL (IQR, 40–80) in the contrast-guided group; contrast was not used in pressure-guided procedures.

The relative reduction in X-ray exposure was similar in patients with BMI ≤ 30 and >30 kg/m^2^, but the absolute reduction was larger in those with BMI > 30 kg/m^2^, making this dose-saving effect particularly important for this group (Table 2b).

Supplemental touch-up ablation using an irrigated-tip RF catheter was infrequent in both groups (five patients in the contrast-guided group and two patients in the pressure-guided group) when cryoablation failed to achieve acute PVI or was stopped prematurely due to the risk of developing phrenic nerve paresis, and a switch to RF was deemed safer in order to complete isolation (Table 3). Given the low absolute number of events, this observation could not be used as a principal measure of procedural efficacy.

Although overall complication rates were similar between the groups (7.8% vs. 7.5%, Table 4), the study was not powered to prove safety equivalence or non-inferiority.

### 3.3. Redo-Procedure Data

The 112 repeat procedures performed for symptomatic arrhythmia recurrence following CB-PVI at our institution (56 patients each from either group) were performed within a time frame of 3 to 55 (mean 17.3 ± 11.7) months following the index ablation.

Among patients undergoing repeat ablation, 174/224 pulmonary veins (78%) remained isolated after pressure-guided procedures, compared with 150/224 (67%) after contrast-guided procedures. These per-vein findings are presented descriptively, without inferential comparison because of within-patient clustering. Right-sided veins accounted for more reconnections than left-sided veins in both groups. During the repeat study, low-voltage areas were identified in 50/112 patients (44.6%): 20/56 (35.7%) in the contrast-guided group and 30/56 (53.6%) in the pressure-guided group (Table 5).

## 4. Discussion

This large single-center observational analysis provides three main findings. First, pressure-waveform guidance for PV occlusion during CB-PVI was feasible in routine practice. Second, pressure-guided procedures were associated with markedly lower intraprocedural fluoroscopy exposure, while procedure duration, ablation time, and acute complication rates were similar. Third, in the selected subset of patients undergoing repeat ablation, pressure-guided index procedures were associated with a higher proportion of durably isolated veins. However, this latter observation should be viewed as a signal associated with pressure-guided treatment, rather than as proof of a causal effect attributable solely to the occlusion-assessment method.

Our findings are in line with the recently published prospective multicenter POLAR-WAVE study [22], which showed good 1-year arrhythmia-free survival using pressure-guided cryoablation in 220 patients. While POLAR-WAVE provided important clinical outcome data, our study adds further perspective by including invasive reassessment during repeat procedures in a subset of patients, giving direct insight into lesion durability. With a larger cohort of 1166 patients and consistent technique across operators, we also offer a real-world perspective on procedural performance. Together, these results complement each other and strengthen the case for pressure-guided assessment as a feasible approach in everyday practice.

### 4.1. Pressure-Guided vs. Contrast-Guided Assessment of Vein Occlusion

In this study, total ablation time and procedure duration did not differ significantly between groups, and, RF touch-up ablation was infrequent in both groups. Because of the small number of events and the different reasons for switching to RF, this endpoint should be interpreted descriptively rather than as evidence of superior procedural efficacy.

In one small study [10] including 18 patients, pressure waveform changes did not reliably predict complete angiographic PV occlusion during CB ablation. In contrast, more recent larger studies [8,13,14] have shown that pressure-guided occlusion assessment is feasible and clinically effective, with comparable outcomes and reduced contrast use.

Several small studies [9,12] have explored pressure curve monitoring as an adjunctive (add-on) strategy. Similarly to our findings, a small prospective randomized study using the second-generation CB [8] reported comparable efficacy with pressure-guided versus conventional (contrast-guided) CB ablation, alongside significant reductions in contrast volume and radiation exposure.

### 4.2. Reduction in X-Ray Exposure

In the pressure-guided group, relying exclusively on real-time pressure curve monitoring without the use of CM eliminated the need for high-quality imaging to assess vein occlusion, leading to a marked reduction in fluoroscopic exposure.

In previous studies, the reduction in fluoroscopy times was greatest when assessment of occlusion was exclusively guided by pressure-curve analysis, whereas use of a combination of contrast- and pressure-guided approaches [9,12] resulted in much smaller reductions of fluoroscopy dose [13], and a number of recent studies reported relatively long (i.e., >20 min) fluoroscopy times [9,12].

Increased fluoroscopy exposure may sometimes be necessary to improve visualization of vein occlusion or contrast leakage [9]. In some cases, multiple contrast injections are necessary to optimize the balloon positioning until adequate occlusion is achieved.

Interpretation of radiation findings requires caution. Preprocedural CT was routinely used in both groups and integrated into the fluoroscopy system as an anatomical guidance during the procedure. Therefore, the observed difference in radiation exposure refers to intraprocedural fluoroscopy exposure. This reduction is particularly relevant for the EP lab staff, for whom radiation exposure is cumulative and repeated on a daily basis, translating into an important long-term occupational benefit.

### 4.3. Redo Procedures

In the subgroup of 112 patients undergoing repeat ablation procedures for symptomatic arrhythmia recurrence, the pressure-guided group suggested a higher rate of durable PVI compared to the contrast-guided group. In small cohorts [23,24,25,26] undergoing repeat ablation for recurrent atrial arrhythmias after CB-PVI, durable isolation of all pulmonary veins was observed in 25–33% of patients, with per-vein durable isolation rates of 63–69% in the two largest series. The higher rate of durable isolation seen in the pressure-guided group should be interpreted as an association, not proof of a causal effect. One possible explanation is that pressure guidance may reflect more accurate occlusion assessment than CM angiography, which can miss subtle leaks or partial occlusion, especially in patients with higher body mass index with limited image quality, since absence of visible contrast leakage does not necessarily indicate adequate balloon–tissue contact. Although these findings are encouraging, our study did not directly assess balloon–tissue contact and therefore cannot definitively establish whether pressure guidance provides a more accurate assessment of occlusion than contrast angiography.

In addition, the chronological adoption of the two strategies complicates causal interpretation, as increasing operator experience and evolving laboratory workflows coincided with the introduction of pressure guidance and probably contributed to the observed procedural differences. Therefore, the observed durability findings should be considered exploratory and hypothesis-generating, representing a potential durability signal associated with pressure guidance rather than evidence of a causal treatment effect.

Consistent with earlier studies [23,27], right-sided veins were more frequently reconnected during repeat procedures, likely reflecting technical and anatomical challenges that can limit coaxial alignment and circumferential balloon contact, particularly with a posterior transseptal puncture that restricts maneuverability and compromises occlusion.

In addition, phrenic nerve pacing during right-sided freezes may reduce balloon–tissue contact and lesion quality, contributing to the difficulty of achieving durable isolation of right-sided pulmonary veins.

Interestingly, low-voltage areas were common during redo procedures (about 45% in our cohort). This is in line with prior data [28], suggesting that both pulmonary vein reconnection and progressive atrial remodeling may contribute to arrhythmia recurrence.

## 5. Limitations

(1) This study is a non-randomized single-center observational study with the inherent limitations of this design. (2) Although all procedures were performed by experienced and well-trained operators, it should be noted that contrast-guided procedures were performed predominantly during the earlier study period, whereas pressure-guided procedures were performed later. Consequently, improvements in operator experience, fluoroscopy practice, and other aspects of the institutional workflow over time may have contributed to the observed differences. Although cases from the initial cryoballoon learning phase were excluded to reduce bias related to early operator experience, the findings should still be interpreted as associations rather than evidence of a causal treatment effect. (3) The use of a single cryoablation catheter platform from one manufacturer may limit the generalizability of these findings to other systems. (4) Redo-procedure findings should be interpreted cautiously, as they were derived from a selected subgroup of patients with recurrent arrhythmia who underwent repeat ablation, while systematic invasive reassessment of pulmonary vein durability was not performed in all patients. Long-term rhythm outcome assessment was further limited by incomplete follow-up and the absence of continuous rhythm monitoring.

## 6. Conclusions

Pressure-guided assessment of vein occlusion during CB-PVI represents a feasible alternative to contrast-guided occlusion assessment and was associated with significantly lower intraprocedural fluoroscopy exposure during the index procedure. Findings from repeat procedures suggest a potential durability signal, but this observation remains hypothesis-generating and requires confirmation in prospective randomized studies with systematic follow-up.

## Figures and Tables

**Figure 1:**
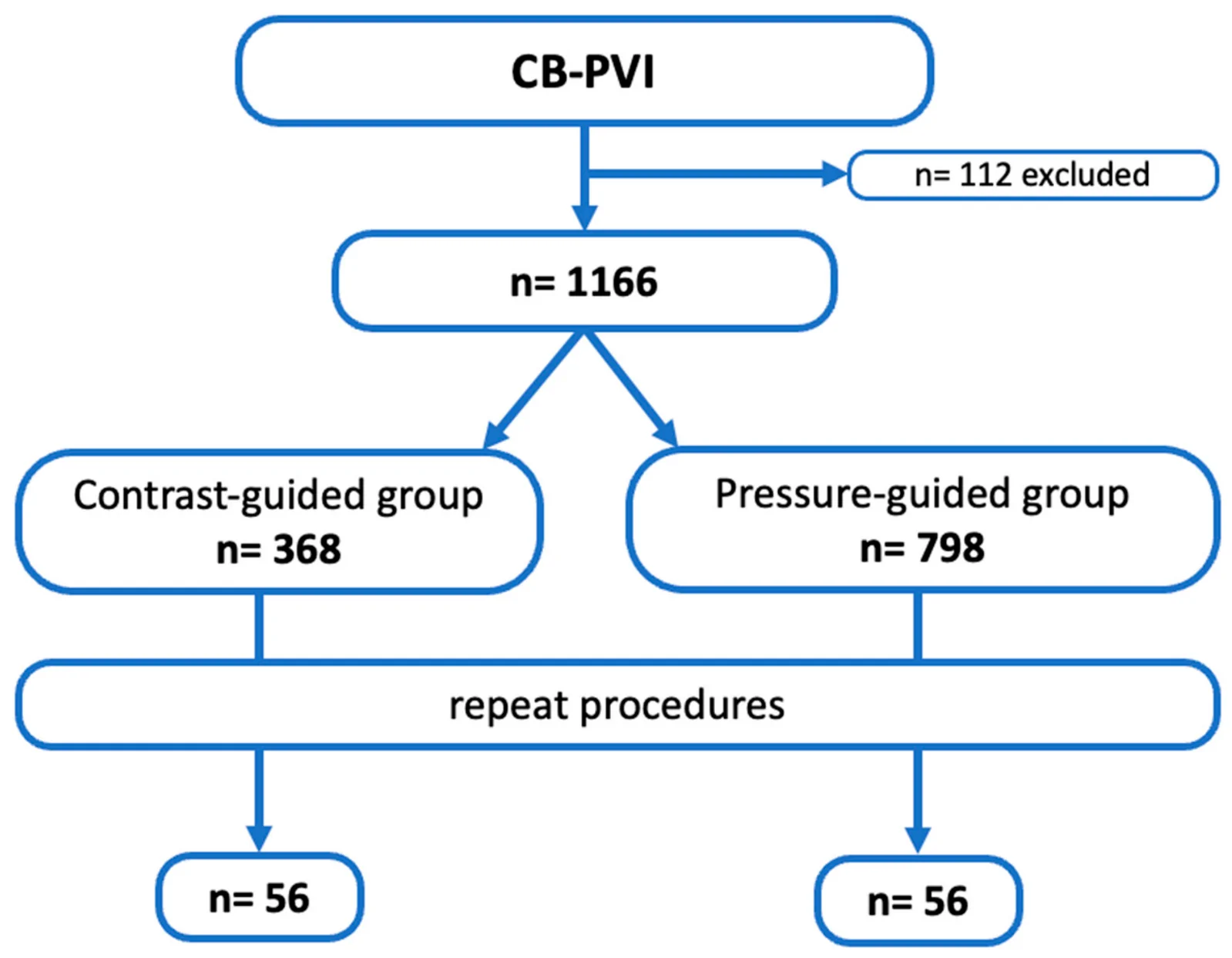
Flowchart of patient selection for first-time CB-PVI procedures between July 2018 and December 2023. After applying exclusion criteria, 1166 patients were included in the analysis: 368 in the contrast-guided group and 798 in the pressure-guided group. The very early cases were not included in this cohort to minimize the potential impact of the initial learning curve. Patients requiring additional ablation or with aborted procedures due to severe complications or technical issues were excluded. A total of 56 repeat procedures were performed in each group. CB-PVI: cryoballoon pulmonary vein isolation.

**Figure 2:**
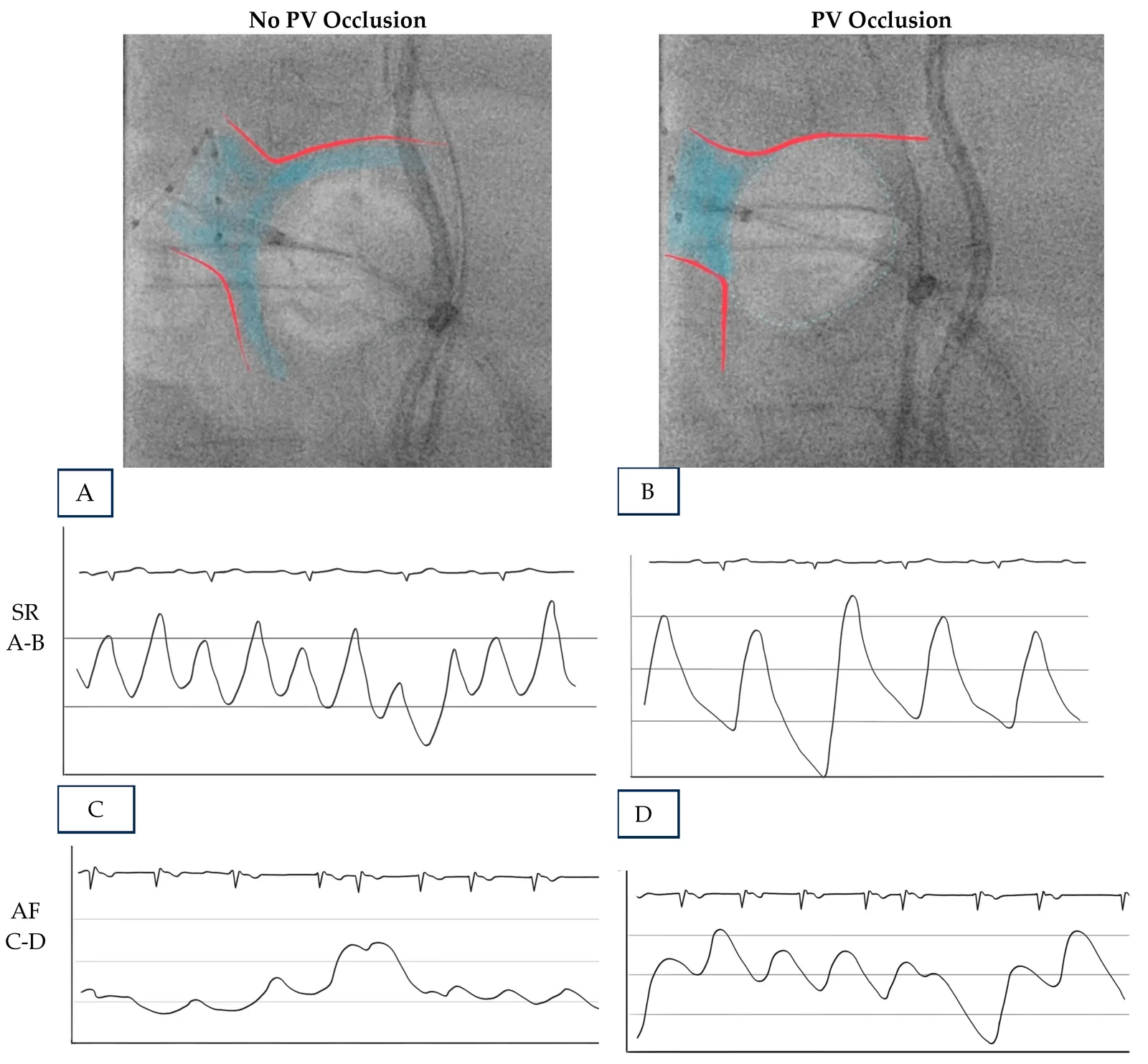
Pressure waveforms recorded from the distal lumen of the cryoballoon catheter with (right panels) and without (left panels) PV occlusion. In the upper fluoroscopic images, red lines outline the PV contours, and blue shading schematically represents contrast distribution. Without occlusion, the tracing reflects left atrial pressure, with A and V waves in sinus rhythm (**A**) and a predominantly V-wave pattern during AF (**C**). Complete PV occlusion is characterized by a transition to a pulmonary artery-like waveform, with an asymmetric contour, steep upstroke, and slower downstroke ((**B**) in SR, (**D**) in AF). AF: atrial fibrillation; PV: pulmonary vein; SR: sinus rhythm.

**Table 1:** (**a**) Baseline patient characteristics. (**b**) Chronological distribution of procedures according to pulmonary vein occlusion-assessment strategy.

(**a**)								
		**Contrast-Guided Group**		**Pressure-Guided Group**	**Unadjusted *p*-Value**	**SMDs**		
						**Unadjusted**	**PS-Matched**	**IPTW**
Number		368 (31.6%)		798 (68.4%)				
Female		154 (41.8%)		331 (41%)	0.95	0.007	0.055	0.001
Persistent AF		91 (25%)		328 (41%)	<0.0001	0.354	0.059	0.059
Age (years)		64.5 ± 10.2		65.7 ± 10.5	0.06	0.119	0.004	0.004
BMI (kg/m^2^)		28.2 ± 4.3		28.1 ± 4.1	0.73	0.021	0.034	0.004
DM		63 (17%)		159 (20%)	0.3	0.072	0.035	0.007
HTN		289 (78%)		656 (82%)	0.15	0.093	0.021	0.019
Dyslipidemia		278 (75%)		640 (80%)	0.08	0.112	0.066	0.010
Thyroid disease		71 (19%)		169 (21%)	0.48	0.047	0.000	0.023
PAD		29 (8%)		67 (8%)	0.82	0.019	0.020	0.014
Previous TIA/Stroke		19 (5%)		57 (7%)	0.25	0.082	0.091	0.021
COPD		26 (7%)		41 (5%)	0.22	0.081	0.045	0.004
GFR < 60 (mL/min/1.73 m^2^)		26 (7%)		63 (8%)	0.72	0.032	0.021	0.007
CAD		67 (18%)		143 (18%)	0.9	0.007	0.014	0.008
CHA_2_DS_2_-VASc (median)		2 [1–3]		3 [1–4]	0.04	0.129	0.038	0.021
EF < 40%		24 (6%)		60 (7%)	0.6	0.039	0.011	0.004
LAV (mL)		116.1 ± 26.9		117.7 ± 28.7	0.37	0.059	0.084	0.036
LAVI (mL/m^2^)		58.7 ± 14		59.4 ± 14.6	0.47	0.048	0.098	0.052
(**b**)								
**Group**	**2018**		**2019**	**2020**	**2021**	**2022**	**2023**	**Total**
Contrast-guided	69		177	114	8	0	0	368
Pressure-guided	0		2	71	231	241	253	798
Total	69		179	185	239	241	253	1166

Values are presented as number (percentage), mean ± standard deviation, or median [25th–75th percentile]. Group summaries and *p*-values refer to the unadjusted cohort. Absolute standardized mean differences (SMDs) describe between-group balance before adjustment (unadjusted), after propensity-score matching (PS-matched), and after inverse probability of treatment weighting (IPTW). Smaller SMDs indicate better balance; values < 0.10 were considered indicative of acceptable balance. AF, atrial fibrillation; BMI, body mass index; CAD, coronary artery disease; COPD, chronic obstructive pulmonary disease; DM, diabetes mellitus; EF, ejection fraction; GFR, glomerular filtration rate; HTN, hypertension; IPTW, inverse probability of treatment weighting; IQR, interquartile range; LAV, left atrial volume; LAVI, left atrial volume index; PAD, peripheral artery disease; PS, propensity score; SMD, standardized mean difference; TIA, transient ischemic attack. Both strategies were used in 16 of 66 calendar months (24.2%).

**Table 2:** (**a**) Procedural outcomes and adjusted comparisons between pressure-guided and contrast-guided ablation. (**b**) X-ray dose by BMI subgroup.

(**a**)					
**Outcome**	**Contrast-Guided (** ***n* = 368)**	**Pressure-Guided (** ***n* = 798)**	**Unadjusted *p*-Value**	**IPTW: Mean Difference (95% CI); *p*-Value**	**PS-Matched: Mean Difference (95% CI); *p*-Value**
Fluoroscopy time, min	9.5 ± 4.8	5.7 ± 3.4	<0.001	−3.92 (−4.48 to −3.37); *p* < 0.001	−3.81 (−4.46 to −3.17); *p* < 0.001
Radiation dose, cGy/cm^2^	585.7 ± 546.6	82.5 ± 78.8	<0.001	−488.48 (−538.72 to −438.25); *p* < 0.001	−505.76 (−562.99 to −448.54); *p* < 0.001
Effective radiation dose, mSv	1.28 ± 1.16	0.19 ± 0.17	<0.001	−1.063 (−1.171 to −0.956); *p* < 0.001	−1.101 (−1.222 to −0.979); *p* < 0.001
Procedure duration, min	92.7 ± 22.7	90.1 ± 20.1	0.057	−2.640 (−5.277 to −0.003); *p* = 0.0497	−2.073 (−5.195 to 1.048); *p* = 0.192
Ablation time, s	1368 ± 406	1342 ± 361	0.283	−25.12 (−74.12 to 23.87); *p* = 0.315	−46.21 (−101.40 to 8.99); *p* = 0.101
(**b**)					
		**BMI > 30**		**BMI** ≤ **30**	
		Contrast-guided	Pressure-guided	Contrast-guided	Pressure-guided
X-ray dose [cGy/cm^2^]		842 ± 777	494 ± 403	118 ± 128	69 ± 56

Group values are unadjusted means ± standard deviations. Mean differences are expressed as pressure-guided minus contrast-guided values; negative differences indicate lower values with pressure guidance. IPTW was the main adjusted analysis, and propensity-score matching was a sensitivity analysis. All *p*-values are two-sided. CI, confidence interval; IPTW, inverse probability of treatment weighting; PS, propensity score.

**Table 3:** RF touch-up ablation during the index procedure.

	Case	PV	Reason for RF Ablation	Ablation Time (s)	
				CB	RF
Contrast-guided group	1	RSPV	PN paresis	762	610
	2	RIPV	Ineffective CB lesions	1522	155
	3	RIPV	Ineffective CB lesions	1809	186
	4	RSPV	PN paresis	1140	299
	5	LSPV	Ineffective CB lesions	1861	96
Pressure-guided group	1	RSPV	PN paresis	634	282
	2	RIPV	Ineffective CB lesions	1252	201

PV: pulmonary vein, RSPV: right superior pulmonary vein, RIPV: right inferior pulmonary vein, LSPV: left superior pulmonary vein, CB: cryoballoon, PN: phrenic nerve, RF: radiofrequency.

**Table 4:** Acute complications related to index procedures.

Complications	Contrast-Guided Group N (%)	Pressure-Guided Group N (%)
Total	29 (7.8%)	60 (7.5%)
Vascular complications	6 (1.6%)	10 (1.2%)
groin hematoma	4 (1%)	6 (0.7%)
AV fistula	2 (0.5%)	3 (0.3%)
Retroperitoneal hematoma	0	1 (0.1%)
Stroke/TIA	1 (0.2%)	4 (0.5%)
Respiratory complications	2 (0.5%)	5 (0.6%)
Aspiration	2 (0.5%)	4 (0.5%)
Hemoptysis	0	1 (0.1%)
PN palsy	11 (2.9%)	30 (3.7%)
Transient PN palsy	10/11 (2.7%)	28/30 (3.5%)
Persistent * PN palsy	1/11 (0.2%)	2/30 (0.2%)
Gastroparesis	1 (0.2%)	0
Cardiac complications	8 (2.1%)	12 (1.5%)
Transient AVB III	1 (0.2%)	0
VT/VF by rapid ventricular pacing	1 (0.2%)	0
ST elevation due to air embolism	2 (0.5%)	7 (0.8%)
Pericarditis/pericardial effusion	4 (1%)	5 (0.6%)

AV fistula: arteriovenous fistula, TIA: transient ischemic attack, PN: phrenic nerve, AVB III: third-degree atrioventricular block, VT: ventricular tachycardia, VF: ventricular fibrillation. * Persistent (>24 h) phrenic nerve palsy.

**Table 5:** Pulmonary vein reconnection at repeat ablation: descriptive findings.

Finding	Overall	Contrast-Guided	Pressure-Guided
Patients undergoing repeat ablation, n	112	56	56
Pulmonary veins assessed, n	448	224	224
Reconnected veins, n/N (%)	125/448 (27.9%)	74/224 (33.0%)	51/224 (22.8%)
Veins remaining isolated, n/N (%)	324/448 (72.1%)	150/224 (67.0%)	174/224 (78%)
LSPV reconnection, n/N (%)	24/112 (21.4%)	14/56 (25.0%)	10/56 (17.9%)
LIPV reconnection, n/N (%)	27/112 (24.1%)	18/56 (32.1%)	9/56 (16.1%)
RIPV reconnection, n/N (%)	37/112 (33.0%)	20/56 (35.7%)	17/56 (30.4%)
RSPV reconnection, n/N (%)	37/112 (33.0%)	22/56 (39.3%)	15/56 (26.8%)
Patients with all four veins reconnected, n/N (%)	0/112 (0%)	0/56 (0%)	0/56 (0%)
Patients with low-voltage areas, n/N (%)	50/112 (44.6%)	20/56 (35.7%)	30/56 (53.6%)

Vein-specific percentages use the number of assessed veins of the corresponding anatomical type. Patient-level percentages use the number of patients undergoing repeat ablation. These observations should not be interpreted as independent evidence of a between-group difference. LIPV: left inferior pulmonary vein, LSPV: left superior pulmonary vein, LVA: low-voltage areas, RIPV: right inferior pulmonary vein, RSPV: right superior pulmonary vein, PV: pulmonary vein.

## Data Availability

The data supporting the findings of this study are available at our center but are not publicly available due to patient privacy and institutional data protection policies. Data are available from the corresponding author upon reasonable request.

## Abbreviations

The following abbreviations are used in this manuscript:

AF	Atrial fibrillation
CB	Cryoballoon
CM	Contrast media
LA	Left atrium
PVI	Pulmonary vein isolation
RF	Radiofrequency
SR	Sinus rhythm
